# Patient Complexity and Bile Duct Injury After Robotic-Assisted vs Laparoscopic Cholecystectomy

**DOI:** 10.1001/jamanetworkopen.2025.1705

**Published:** 2025-03-25

**Authors:** Cody Lendon Mullens, Sarah Sheskey, Jyothi R. Thumma, Justin B. Dimick, Edward C. Norton, Kyle H. Sheetz

**Affiliations:** 1 Department of Surgery, University of Michigan, Ann Arbor; 2 Center for Healthcare Outcomes and Policy, Institute of Health Policy and Innovation, University of Michigan, Ann Arbor; 3 UM National Clinician Scholars Program, University of Michigan, Ann Arbor; 4 Department of Health Management and Policy, University of Michigan, Ann Arbor; 5 Department of Economics, University of Michigan, Ann Arbor

## Abstract

**Importance:**

Recent evidence suggests higher bile duct injury rates for patients undergoing robotic-assisted cholecystectomy compared with laparoscopic cholecystectomy. Proponents of the robotic-assisted approach contend that this may be due to selection of higher-risk and more complex patients being offered robotic-assisted cholecystectomy.

**Objective:**

To evaluate the comparative safety of robotic-assisted cholecystectomy and laparoscopic cholecystectomy among patients with varying levels of risk for adverse postoperative outcomes.

**Design, Setting, and Participants:**

This retrospective cohort study assessed fee-for-service Medicare beneficiaries aged 66 to 99 years who underwent cholecystectomy between January 1, 2010, and December 31, 2021. Data analysis was performed between June and August 2024. Medicare beneficiaries were separated into model training and experimental cohorts (60% and 40%, respectively). Random forest modeling and least absolute shrinkage and selection operator techniques were then used in a risk model training cohort to stratify beneficiaries based on their risk of a composite outcome of postoperative adverse events consisting of 90-day postoperative complications, serious complications, reoperations, and rehospitalization in an independent experimental cohort.

**Exposures:**

Robotic-assisted vs laparoscopic cholecystectomy.

**Main Outcomes and Measures:**

The primary outcome of interest was bile duct injury requiring operative intervention after cholecystectomy. Secondary outcomes were composite outcomes from cholecystectomy composed of any complications, serious complications, reoperations, and readmissions.

**Results:**

A total of 737 908 individuals (mean [SD] age, 74.7 [9.9] years; 387 563 [52.5%] female) were included, with 295 807 in an experimental cohort and 442 101 in a training cohort. Bile duct injury was higher among patients undergoing robotic-assisted compared with laparoscopic cholecystectomy in each subgroup (low-risk group: relative risk [RR], 3.14; 95% CI, 2.35-3.94; medium-risk group: RR, 3.13; 95% CI, 2.35-3.92; and high-risk group: RR, 3.11; 95% CI, 2.34-3.88). Overall, composite outcomes between the 2 groups were similar for robotic-assisted cholecystectomy compared with laparoscopic cholecystectomy (RR, 1.09; 95% CI, 1.07-1.12), aside from reoperation, which was overall higher in the robotic-assisted group compared with the laparoscopic group (RR, 1.47; 95% CI, 1.35-1.59).

**Conclusions and Relevance:**

In this cohort study of Medicare beneficiaries, bile duct injury rates were higher among low-, medium-, and high-risk surgical candidates after robotic-assisted cholecystectomy. These findings suggest that patient selection may not be the cause of differences in bile duct injury rates among patients undergoing robotic-assisted vs laparoscopic cholecystectomy.

## Key Points

**Question:**

Are patient risk factors and complexity associated with differential rates of bile duct injury in robotic-assisted vs laparoscopic cholecystectomy?

**Findings:**

In this cohort study of 737 908 Medicare beneficiaries, bile duct injury rates were 3 times higher for patients who underwent robotic-assisted cholecystectomy compared with laparoscopic cholecystectomy across the spectrum of patient risk factors. Significantly higher rates of bile duct injury were found in low-risk patients who underwent robotic-assisted cholecystectomy compared with high-risk patients who underwent laparoscopic cholecystectomy.

**Meaning:**

These findings suggest that while overall outcomes are similar, bile duct injury rates are uniformly higher for robotic-assisted than for laparoscopic cholecystectomy regardless of patient complexity.

## Teaser

This cohort study examines the comparative safety of robotic-assisted vs laparoscopic cholecystectomy according to patient risk factors.

## Introduction

In the previous decade, robotic-assisted cholecystectomy has increased 37-fold.^1^ Recent evidence has raised concerns for higher rates of bile duct injury in patients undergoing robotic-assisted cholecystectomy compared with laparoscopy.^1^ Bile duct injury is a technical complication associated with high levels of morbidity and health care costs.^2,3,4^ One potential mechanism contributing to the increased risk of bile duct injury in robotic-assisted cholecystectomy could be related to its use as a training case for credentialing in the context of its long learning curve.^5^ However, proponents of robotic-assisted cholecystectomy suggest that this approach may be beneficial in higher-risk cases to avoid open cholecystectomies.^6,7^

Whether robotic-assisted cholecystectomy offers an advantage over laparoscopic cholecystectomy for higher-risk cases remains unclear. On one hand, there may be fundamental differences in the complexity of patients undergoing robotic-assisted cholecystectomy, which may be responsible for the higher observed rates of bile duct injury. On the other hand, differences in bile duct injury could be secondary to other factors, such as surgeons working their way up the learning curve using the robot, especially given the large number of robotic-assisted cholecystectomies surgeons must perform to achieve bile duct injury rates equivalent to those of laparoscopic approaches.^5^ By comparing laparoscopic and robotic-assisted cholecystectomy approaches within patient risk terciles, we can determine whether patient risk factor profiles are associated with harm in robotic-assisted cholecystectomy.

The aim of this study was to examine the comparative safety of robotic-assisted vs laparoscopic cholecystectomy stratified based on patient risk factors. We accomplished this using Medicare data to devise a model to stratify patients based on risk factors for adverse outcomes from cholecystectomy. In doing this, we were able to compare outcomes, such as bile duct injury, from robotic-assisted and laparoscopic cholecystectomy within patient risk stratifications.

## Methods

This cohort study was acknowledged as being exempt from review by the University of Michigan Institutional Review Board, which waived the need for patient informed consent because of the deidentified data used in this study. Our study followed the Strengthening the Reporting of Observational Studies in Epidemiology (STROBE) guideline.^8^ Analysis of these data took place between June and August 2024.

### Data Source and Study Population

We used Medicare Provider Analysis and Review (MedPAR) data for acute care hospitals’ inpatient admission claims from January 1, 2010, to December 31, 2021, to identify patients undergoing cholecystectomy using *International Classification of Diseases, Ninth Revision* (*ICD-9*) and *International Statistical Classification of Diseases and Related Health Problems, Tenth Revision* (*ICD-10*), and *Current Procedural Technology* codes (eTable 1 in Supplement 1). We identified laparoscopic vs robotic-assisted cholecystectomy using previously described methods.^1,9,10^ We included patients who were aged 66 to 99 years and were continuously enrolled in Medicare fee for service with no managed care enrollment for 3 months before and 12 months after their index operation. We excluded patients with documented diagnosis of gallbladder, bile duct, or liver malignant neoplasm. We linked American Hospital Association survey data by using Medicare National Provider Identifier to define hospital characteristics. Using the Elixhauser method, we collected patient demographic data and comorbidities using *ICD-9* and *ICD-10* diagnosis codes.^11^

Race and ethnicity data, along with other patient characteristics, were obtained from MedPAR data. Race and ethnicity categories available in these data included Asian, Black, Hispanic, North American Native, White, other, and unknown. No specific categories of race or ethnicity in the “other” group are reported specifically by Medicare claims. We collected data on race and ethnicity for this study to identify and account for potential racial and ethnic disparities and assess the generalizability of our study.

### Outcomes and Explanatory Variables

Our primary outcome was bile duct injury requiring operative repair with hepaticojejunostomy or choledochojejunostomy within 1 year of a beneficiary’s index cholecystectomy. We identified these severe bile duct injuries requiring operative intervention using *ICD-9* and *ICD-10* codes for bile duct injury and codes for operative repair with hepaticojejunostomy or choledochojejunostomy (eTable 1 in Supplement 1).

Adverse postoperative outcomes (eg, complications, reoperations, and readmission) are more likely to occur in patients who have higher levels of preoperative risk. We created a 90-day composite outcome score by incorporating the incidence of any complications, serious complications, reoperations, and rehospitalization for any cause after index cholecystectomy, with values ranging from 0 (for none of these outcomes) to 4 (for all 4 postoperative outcomes).^12,13,14^ Serious complications were defined as complications with a hospital length of stay greater than the 75th percentile.^15^ Explanatory variables used in our risk-adjusted modeling for bile duct injury rates, described later, included patient age, sex, race and ethnicity, Elixhauser comorbidities, indication for an operation (eg, cholecystitis), year of surgery, and surgical approach (robotic-assisted or laparoscopic).

### Statistical Analysis

The primary goal of this analysis was to determine whether outcomes between robotic-assisted and laparoscopic cholecystectomy differ among patients with varying levels of risk for adverse postoperative events. We randomly divided our retrospective cohort dataset into 2 samples: 60% for a training cohort and 40% for an experimental cohort. The training cohort was used to identify and prioritize the most influential patient and hospital factors associated with a 90-day composite outcome score. To achieve this, we used a 2-step feature selection process. We used a random forest algorithm to rank the importance of a comprehensive set of variables, including patient characteristics (age, sex, race, primary diagnosis, total number of diagnoses, total number of procedures, hospital length of stay associated with the index operation, 29 Elixhauser comorbidities, year of surgery, and type of admission) as well as hospital characteristics (number of operating rooms, registered nurse to census ratio, profit status, bed size, teaching status, rural vs urban classification, and geographic region). To optimize the random forest model, we performed a randomized search over a range of hyperparameters using RandomizedSearchCV from the scikit-learn library.^16^ This process involved 10 iterations with 3-fold cross-validation within the training cohort to identify the best combination of hyperparameters that maximized model accuracy. Use of this modeling approach using random forest algorithms allowed us to capture more complex associations compared with pure linear associations.

After the identification of key variables with the optimized random forest model, we applied the least absolute shrinkage and selection operator (LASSO) technique using 10-fold cross-validation to further refine the selection of predictors. LASSO was implemented to narrow the set of variables by reducing the influence of less important variables, focusing on those most strongly associated with the outcome. The final set of variables identified through LASSO was then used in a Poisson regression model, appropriate for modeling the count-based 90-day composite outcome score. We validated the model by applying it to the experimental cohort and assessed its reliability and robustness. The model’s performance was evaluated using root mean square error, which was 0.85, indicating that the model’s predictions were a mean of 0.85 units away from the actual observed values. Given the outcome range of 0 to 4, this suggests that the model’s predictions were typically within 1 unit of the true values.

We then calculated predicted patient risk score for 90-day composite outcome scores using the selected covariates and divided the score into terciles to stratify patients into low-, medium-, and high-risk groups. We then compared bile duct injury rate and individual components of 90-day complications: any complications, serious complications, reoperations, and rehospitalizations by surgical approach and patient risk group terciles. All analyses were performed using SAS software, version 9.4 (SAS Institute Inc) and Stata software, version 18 (StataCorp LLC). Tests were 2-sided, and significance was set at *P* < .05 or 95% CIs excluding 1.

## Results

### Patient Characteristics

A total of 737 908 individuals (mean [SD] age, 74.7 [9.9] years; 387 563 [47.5%] female and 350 345 [57.4%] male) participated in the study. Patient characteristics were similar between the training cohort (n = 442 101) and the experimental cohort (n = 295 807). The percentages of patients with Elixhauser comorbidities were similar across the 2 cohorts. Cholecystitis was the most common indication for cholecystectomy for both the training cohort (388 254 [87.8%]) and the experimental cohort (259 082 [87.6%]) (*P* = .003). Table 1 and eTable 2 in Supplement 1 outline additional patient characteristics whose mean values were similar between the training and experimental cohorts. Additional patient demographics for the experimental cohort are given in eTables 3 to 6 in Supplement 1, which compare patient demographics by risk tercile based on operative approach. Robotic-assisted cholecystectomy comprised 3.2% (n = 14 252) and 3.7% (n = 10 980) of the training and experimental cohort, respectively (*P* < .001). Hospital characteristics compared between the training and experimental cohort are also summarized in Table 1.

**Table 1.** Comparison of Patient and Hospital Characteristics for Beneficiaries in Training and Experimental Cohorts Who Underwent Cholecystectomy

Characteristic	No. (%)^a^		*P* value
	Training cohort (n = 442 101)	Experimental cohort (n = 295 807)	
**Patient characteristics**			
Age, mean (SD), y	74.7 (9.8)	74.7 (9.9)	.07
Sex			
Female	232 367 (52.6)	155 196 (52.5)	.43
Male	209 734 (47.4)	140 611 (47.5)	
Race and ethnicity			
Asian	7679 (1.7)	5488 (1.9)	<.001
Black	31 156 (7.0)	20 849 (7.0)	
Hispanic	12 827 (2.9)	8283 (2.8)	
North American Native	3364 (0.8)	2252 (0.8)	
White	374 883 (84.8)	250 895 (84.8)	
Other^b^	7644 (1.7)	4941 (1.7)	
Unknown	4548 (1.0)	3099 (1.0)	
Length of stay, mean (SD), d	5.4 (5.6)	5.4 (5.5)	.08
No. of diagnoses during admission, mean (SD)	12.6 (6.2)	12.6 (6.2)	.63
No. of surgical procedure count, mean (SD)	2.7 (2.0)	2.7 (2.0)	.01
No. of Elixhauser comorbidities			
0	27 350 (6.2)	18 455 (6.2)	.49
1	70 767 (16.0)	47 930 (16.2)	
2	95 711 (21.6)	63 772 (21.6)	
3	88 477 (20.0)	59.363 (20.1)	
4	67 043 (15.2)	44 407 (15.0)	
≥5	92 753 (21.0)	61 880 (20.9)	
Indication for cholecystectomy			
Cholecystitis	388 254 (87.8)	259 082 (87.6)	.003
Cholangitis	18 819 (4.3)	12 758 (4.3)	.24
Cholelithiasis	29 043 (6.6)	19 696 (6.7)	.13
Other	72 002 (16.3)	48 257 (16.3)	.76
Approach			
Robotic	14 252 (3.2)	10 980 (3.7)	<.001
Laparoscopic	427 849 (96.8)	284 827 (96.3)	<.001
90-d Composite adverse outcome components			
Any complications	114 902 (26.0)	76 845 (26.0)	.91
Serious complications	47 206 (10.7)	31 414 (10.6)	.43
Reoperations	15 757 (3.6)	10 589 (3.6)	.72
Readmissions	87 502 (19.8)	58 769 (19.9)	.43
**Hospital characteristics**			
Profit status			
For profit	74 372 (16.8)	46 431 (15.7)	<.001
Nonprofit	320 154 (72.4)	219 549 (74.2)	
Other profit	45 484 (10.3)	28 504 (9.6)	
Missing	2091 (0.5)	1323 (0.4)	
Bed capacity			
<250	189 716 (42.9)	122 706 (41.5)	<.001
250-499	156 046 (35.3)	108 075 (36.5)	
≥500	94 248 (21.3)	63 703 (21.5)	
Missing	2091 (0.5)	1323 (0.4)	
Teaching hospital	273 792 (61.9)	184 287 (62.3)	.002
Region			
Midwest	100 268 (22.7)	64 200 (21.7)	<.001
Northeast	68 587 (15.5)	46 479 (15.7)	
South	189 346 (42.8)	126 733 (42.8)	
West	81 809 (18.5)	57 072 (19.3)	
Missing	2091 (0.5)	1323 (0.4)	
Overall inpatient surgical volume, mean (SD)	4918.9 (4969.0)	5073.9 (5248.3)	<.001
No. of operating rooms, mean (SD)	19.7 (18.6)	20.2 (18.9)	<.001
Nurse to census ratio, mean (SD)	8.5 (3.4)	8.6 (3.3)	<.001

^a^ Unless otherwise indicated.

^b^ No specific categories of race or ethnicity in the “other” group are reported specifically by Medicare claims.

### Bile Duct Injury

Using risk groups based on composite outcome scores, we assessed bile duct injury rates among the patient population subdivided by operative approach (Table 2; eTable 7 in Supplement 1). In the experimental cohort, mean bile duct injury rates were overall higher (mean rate, 0.72 [95% CI, 0.55-0.89] vs 0.23 [95% CI, 0.21-0.25]) among patients undergoing robotic-assisted cholecystectomy compared with laparoscopic cholecystectomy (relative risk [RR], 3.12; 95% CI, 2.34-3.91). Similar trends were identified in the training cohort. Furthermore, rates of bile duct injury increased for both surgical approaches across patient risk stratification. When comparing bile duct injury rates in the experimental cohort across operative approach, we found that bile duct injury was higher among patients undergoing robotic-assisted compared with laparoscopic cholecystectomy in the low-risk group (mean rate, 0.47 [95% CI, 0.34-0.60] vs 0.15 [95% CI, 0.13-0.17]; RR, 3.14; 95% CI, 2.35-3.94), medium-risk group (mean rate, 0.66 [95% CI, 0.49-0.84] vs 0.21 [95% CI, 0.18-0.24]; RR, 3.13; 95% CI, 2.35-3.92), and high-risk group (mean rate, 1.03 [95% CI, 0.79-1.28] vs 0.33 [95% CI, 0.30-0.37]; RR, 3.11; 95% CI, 2.34-3.88) (Table 2 and Figure). Of note, in the experimental cohort, the bile duct injury rate for patients undergoing laparoscopic cholecystectomy in the high-risk group (mean rate, 0.33; 95% CI, 0.30-0.37) was lower than the bile duct injury rate for those undergoing robotic-assisted cholecystectomy in the low-risk group (mean rate, 0.47; 95% CI, 0.34-0.60).

**Table 2.** Bile Duct Injury Rates in the Experimental Cohort, Stratified by Operative Approach and Patient Risk Factor Terciles

Risk group	Mean rate (95% CI)		Relative risk (95% CI)
	Robotic-assisted cholecystectomy	Laparoscopic cholecystectomy	
Low	0.47 (0.34-0.60)	0.15 (0.13-0.17)	3.14 (2.35-3.94)
Medium	0.66 (0.49-0.84)	0.21 (0.18-0.24)	3.13 (2.35-3.92)
High	1.03 (0.79-1.28)	0.33 (0.30-0.37)	3.11 (2.34-3.88)
Overall	0.72 (0.55-0.89)	0.23 (0.21-0.25)	3.12 (2.34-3.91)

**Figure.**
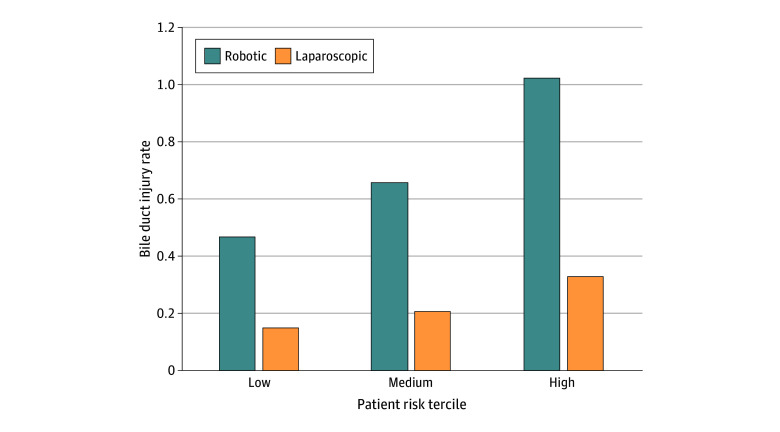
Differences in Bile Duct Injury After Cholecystectomy Within Our Experimental Cohort Among Medicare Beneficiaries, Stratified by Operative Approach and Patient Risk Tercile

### Composite Outcomes

Composite outcome scores in the training and experimental cohorts stratified by operative approach and patient risk are summarized in Table 3 (experimental cohort) and eTable 8 in Supplement 1 (training cohort). We also compared outcome rates that were included in our composite outcome score for the training (eTable 9 in Supplement 1) and experimental (Table 4) cohorts. Overall, outcomes between the robotic-assisted and laparoscopic cholecystectomy experimental cohorts were similar for the composite outcome (RR, 1.09; 95% CI, 1.07-1.12), any complication (RR, 1.11; 95% CI, 1.08-1.15), serious complication (RR, 1.16; 95% CI, 1.11-1.22), and readmission (RR, 1.02; 95% CI, 0.98-1.06). Reoperation rates were higher among patients undergoing robotic-assisted cholecystectomy (RR, 1.47; 95% CI, 1.35-1.59), consistent with the observed rate of bile duct injury rates and our coding strategy to capture reoperations for bile duct injury.

**Table 3.** Ninety-Day Composite Adverse Outcome Scores Within the Experimental Cohort Stratified by Operative Approach and Patient Risk Factor Terciles

Risk group	Mean score (95% CI)	
	Robotic-assisted cholecystectomy	Laparoscopic cholecystectomy
Low	0.20 (0.19-0.21)	0.17 (0.16-0.18)
Medium	0.50 (0.49-0.51)	0.49 (0.48-0.50)
High	1.18 (1.16-1.19)	1.13 (1.12-1.14)
Overall	0.71 (0.70-0.72)	0.60 (0.59-0.61)

**Table 4.** Overall Rates of Discrete Outcomes in the Experimental Cohort Based on Patient Risk Stratification and Operative Approach for Cholecystectomy Among Medicare Beneficiaries

90-d Outcomes	Mean rate (95% CI)		Relative risk (95% CI)
	Robotic-assisted cholecystectomy	Laparoscopic cholecystectomy	
Low risk			
Composite outcome	15.50 (14.11-16.90)	16.01 (15.78-16.24)	0.97 (0.88-1.06)
Any complications	6.05 (5.13-6.97)	6.34 (6.19-6.50)	0.95 (0.81-1.10)
Serious complications	0.31 (0.10-0.52)	0.80 (0.74-0.85)	0.39 (0.12-0.66)
Reoperations	2.71 (2.09-3.34)	1.84 (1.76-1.93)	1.47 (1.12-1.82)
Readmissions	10.00 (8.84-11.16)	11.43 (11.23-11.64)	0.87 (0.77-0.98)
Medium risk			
Composite outcome	29.49 (28.03-30.94)	31.70 (31.40-31.99)	0.93 (0.88-0.98)
Any complications	16.15 (14.97-17.32)	18.82 (18.57-19.07)	0.86 (0.79-0.92)
Serious complications	1.80 (1.38-2.22)	2.32 (2.23-2.42)	0.77 (0.59-0.96)
Reoperations	4.84 (4.16-5.53)	3.22 (3.11-3.33)	1.50 (1.28-1.72)
Readmissions	14.98 (13.84-16.12)	17.59 (17.34-17.83)	0.85 (0.79-0.92)
High risk			
Composite outcome	64.10 (62.71-65.48)	64.68 (64.37-64.99)	0.99 (0.97-1.01)
Any complications	52.07 (50.62-53.52)	53.03 (52.71-53.35)	0.98 (0.95-1.01)
Serious complications	27.65 (26.35-28.94)	28.89 (28.60-29.18)	0.96 (0.91-1.00)
Reoperations	6.86 (6.13-7.59)	5.53 (5.39-5.68)	1.24 (1.10-1.38)
Readmissions	30.24 (28.91-31.57)	30.79 (30.49-31.08)	0.98 (0.94-1.03)
Overall			
Composite outcome	40.67 (39.76-41.59)	37.26 (37.08-37.44)	1.09 (1.07-1.12)
Any complications	28.82 (27.97-29.66)	25.87 (25.71-26.03)	1.11 (1.08-1.15)
Serious complications	12.30 (11.68-12.91)	10.56 (10.44-10.67)	1.16 (1.11-1.22)
Reoperations	5.18 (4.77-5.60)	3.52 (3.45-3.59)	1.47 (1.35-1.59)
Readmissions	20.19 (19.44-20.94)	19.85 (19.71-20.00)	1.02 (0.98-1.06)

## Discussion

This cohort study of Medicare beneficiaries comparing robotic-assisted and laparoscopic cholecystectomy outcomes based on patient risk factor stratification had 3 principal findings. First, our risk prediction model, based on patient-level variables observable at the time of the operation, accurately stratified patients in the experimental cohort based on their overall risk of adverse postoperative events. There are multiple reasons to believe that this risk score represents more difficult operations, including the indication of operation (eg, cholecystitis and associated active inflammation) and that patients with a higher burden of comorbidities tend to be those who have delayed presentation and sustain delays in care, which leads to chronic sequelae of biliary pathophysiology that can make cholecystectomy more difficult. Second, among all 3 risk groups (low, medium, and high), bile duct injury rates were 3-fold higher after robotic-assisted cholecystectomy compared with traditional laparoscopic cholecystectomy. Third, rates of other adverse postoperative events (eg, complications or readmissions) were similar among those undergoing robotic-assisted cholecystectomy and those undergoing the laparoscopic procedure. Taken together, the stratification by patient risk factors in this study revealed evidence of higher rates of harm (including bile duct injury) across terciles of patient risk factors when compared with laparoscopic cholecystectomy. An important strength of our modeling approach is that to overturn these findings, there would need to be an omitted variable unrelated to any other data point in our model that is a strong enough contributing factor to diminish the observed 3-fold increase in rates of bile duct injury across patient risk factors, which we have a low suspicion for given that the model accounts for patient characteristics, comorbidities, and cholecystectomy indication.

The findings in this study comparing robotic-assisted and laparoscopic cholecystectomy have important implications for the increasing body of evidence comparing surgical approaches for cholecystectomy. Early, smaller, single-center evaluations comparing robotic-assisted and laparoscopic cholecystectomy revealed similar quality but higher costs associated with robotic-assisted approaches.^17,18^ A larger study of the national inpatient sample revealed higher rates of overall complications after robotic-assisted cholecystectomy compared with laparoscopic cholecystectomy but may have been confounded by patient complexity.^19^ A recent meta-analysis published in a robotic-assisted surgery–centric journal discounted concerns for potential risk of bile duct injury associated with robotic-assisted cholecystectomy.^20^ However, this study did not include the largest study to date comparing safety in approach for cholecystectomy in a large, nationally representative sample, which did reveal evidence of higher rates of bile duct injury.^1^ Criticisms of existing work that has raised concerns for higher rates of bile duct injury in robotic-assisted cholecystectomy were that the study did not account for the claim that many surgeons may choose the robotic-assisted platform for cholecystectomy in more complex patients and cases. Our findings clarify this gap, which revealed that across terciles of patient risk, including low-risk patients, bile duct injury appears to be higher in robotic-assisted cholecystectomy. In fact, low-risk patients who underwent robotic-assisted cholecystectomy had higher rates of bile duct injury than high-risk patients who underwent laparoscopic cholecystectomy.

Our study has important implications for adoption of robotic-assisted surgery and its use in cholecystectomy. The data here add to an emerging body of evidence demonstrating harm associated with robotic-assisted cholecystectomy. Established surgeons undertaking training and proctoring to adopt robotic-assisted surgery and current general surgery trainees understandably use familiar operations to learn to use the robotic-assisted platform before its use in more complex cases. These operations include inguinal hernia repair and cholecystectomy.^21^ However, recent evidence revealed that to obtain equivalent rates of bile duct injuries in robotic-assisted cholecystectomy and in laparoscopic cholecystectomy, between 300 and 450 cases are necessary.^5^ These data, combined with our findings that further underscore the evidence of harms associated with robotic-assisted cholecystectomy, suggest that surgeons should consider leveraging other familiar operations as training cases for becoming comfortable with the robotic-assisted platform. Such training is especially important in the context that robotic-assisted cholecystectomy was associated with higher rates of bile duct injury, even in low-risk patients. Neglecting to consider leveraging other familiar operations as training cases aside from cholecystectomy likely puts patients at higher risks of bile duct injury than if they were to undergo laparoscopic cholecystectomy, unless their surgeon has extensive experience with robotic-assisted cholecystectomy.

Assuming surgeons are going to continue leveraging robotic-assisted platforms for cholecystectomy, the natural question becomes: even with higher rates of bile duct injury in robotic-assisted cholecystectomy, are there other reasons to consider adopting robotic-assisted cholecystectomy? There are likely related factors between hospitals and patients that play a role. For example, hospitals can leverage robotic-assisted surgery as a marketing tool to attract patients to obtain health care services at their facility, contributing to clinical volume and revenue.^22,23,24,25^ At the patient level, patients in need of an operation may have a preference to undergo the procedure with a robotic-assisted approach as opposed to laparoscopic or open surgery, although these data are mixed.^25,26,27,28^ For surgeons, there is emerging evidence suggesting that robotic-assisted platforms have important benefits for improving surgeon ergonomics.^29,30,31^ Additionally, surgeons operating at facilities that lack trainees or adequate technical assistants to actively assist with the operation may stand to benefit from robotic-assisted approaches. Specifically, they may benefit from the additional working arm to assist with retraction and exposure, as well as the ease of camera manipulation when using the robotic-assisted platform. However, at the hospital level, robotic-assisted cases are not reimbursed differently than laparoscopic cases, subjecting the hospitals to the increased costs associated with robotic-assisted surgery. Additionally, the extent to which patients understand the fundamental differences between robotic-assisted and laparoscopic surgery and its potential risks and benefits remains unclear.^32^ The decision to adopt robotic-assisted surgical platforms requires a thoughtful balance of patient safety and advancing clinical practice through adoption of new technologies.

As use of robotic-assisted surgery continues to expand, it must be done with caution and intentional balance of adequately training surgeons on the platform while ensuring patient safety. Rightsizing robotic-assisted surgery for operations where there are clearer benefits for patient outcomes is necessary. In the case of robotic-assisted cholecystectomy, our study provides further evidence of adverse outcomes and bile duct injury from robotic-assisted cholecystectomy across the terciles of low-, medium-, and high-risk patients.

### Limitations

This study has important limitations. First, the administrative claims database of Medicare beneficiaries inherently relies on accurate coding of patient data. Thus, we did not include conversions to an open procedure in this study because these data are not able to be accurately tracked in a high-fidelity manner in contemporary Medicare claims data. However, we used data points and outcomes that are reliably coded in administrative claims data using established methods to assess these covariates and outcomes. Second, we generated our model to stratify patients into terciles based on patient risk factors and did not account for surgeon experience. However, existing evidence reveals that most surgeons (87%) perform robotic-assisted cholecystectomy at relatively low volume (<10 cases).^5^ Third, our model for stratifying patients into risk terciles may be limited in appropriately attributing patient risk to potential for adverse outcomes. However, we included a multitude of patient risk factors that are known to be associated with postoperative complications. Furthermore, our model is based on data points that surgeons would have in hand at the time of preoperative evaluation to influence decision-making in the operative approach for cholecystectomy. Although technical risk (eg, risk of bile duct injury) is challenging to predict, it is also unlikely to exist in isolation. Specifically, it would be unlikely that low-risk patients in this cohort have disproportionate complexity with respect to their gallbladder pathophysiology or anatomy.

## Conclusions

This cohort study of Medicare beneficiaries found that bile duct injuries were more common after robotic-assisted compared with laparoscopic cholecystectomy regardless of patients’ baseline risk. These findings call into question claims that patient selection may be the cause of differences in bile duct injury rates among patients undergoing robotic-assisted vs laparoscopic cholecystectomy.

## Notes

Supplement 1. **eTable 1.** Coding strategy for covariates in our primary analysis. Note: Gallstone pancreatitis included any record with any discharge diagnosis code for gallstones (574* or 575* with any extension) and a concomitant diagnosis of acute pancreatitis (577.0) **eTable 2.** Individual comorbidities in the training and experimental cohorts among Medicare beneficiaries who underwent laparoscopic or robotic cholecystectomy **eTable 3.** Overall patient characteristics in experimental cohort based on operative approach **eTable 4.** Low-risk patient characteristics in experimental cohort based on operative approach **eTable 5.** Medium-risk patient characteristics in experimental cohort based on operative approach **eTable 6.** High-risk patient characteristics in experimental cohort based on operative approach **eTable 7.** Bile duct injury rates in training cohort, stratified by operative approach and patient risk factor terciles as described in methods (low, medium, high risk). Relative risks comparing robotic-assisted versus laparoscopic cholecystectomy **eTable 8.** Rates of 90-day composite adverse outcomes (comprised of any complications, serious complications, reoperations, and readmissions) within training cohort and stratified by operative approach and patient risk factor terciles as described in methods (low, medium, high risk) **eTable 9.** Overall rates of composite outcome measures in training cohort based on patient risk stratification and operative approach for cholecystectomy among Medicare beneficiaries

Supplement 2.
Data Sharing Statement
