# Supplementary material for: Patient Complexity and Bile Duct Injury After Robotic-Assisted vs Laparoscopic Cholecystectomy
Source: JAMA Netw Open. 2025 Mar 25;8(3):e251705. doi: 10.1001/jamanetworkopen.2025.1705 (PMC11937934; doi:10.1001/jamanetworkopen.2025.1705)
Supplement: Supplement 1. — eTable 1. Coding strategy for covariates in our primary analysis. Note: Gallstone pancreatitis included any record with any discharge diagnosis code for gallstones (574* or 575* with any extension) and a concomitant diagnosis of acute pancreatitis (577.0) eTable 2. Individual comorbidities in the training and experimental cohorts among Medicare beneficiaries who underwent laparoscopic or robotic cholecystectomy eTable 3. Overall patient characteristics in experimental cohort based on operative approach eTable 4. Low-risk patient characteristics in experimental cohort based on operative approach eTable 5. Medium-risk patient characteristics in experimental cohort based on operative approach eTable 6. High-risk patient characteristics in experimental cohort based on operative approach eTable 7. Bile duct injury rates in training cohort, stratified by operative approach and patient risk factor terciles as described in methods (low, medium, high risk). Relative risks comparing robotic-assisted versus laparoscopic cholecystectomy eTable 8. Rates of 90-day composite adverse outcomes (comprised of any complications, serious complications, reoperations, and readmissions) within training cohort and stratified by operative approach and patient risk factor terciles as described in methods (low, medium, high risk) eTable 9. Overall rates of composite outcome measures in training cohort based on patient risk stratification and operative approach for cholecystectomy among Medicare beneficiaries [file jamanetwopen-e251705-s001.pdf]

Supplemental Online Content

Mullens CL, Sheskey S, Thumma JR, Dimick JB, Norton EC, Sheetz KH. Patient complexity and bile duct injury after robotic-assisted vs laparoscopic cholecysectomy. *JAMA Netw Open*. Published online March 25, 2025. doi:10.1001/jamanetworkopen.2025.1705

eTable 1. Coding strategy for covariates in our primary analysis. Note: Gallstone pancreatitis included any record with any discharge diagnosis code for gallstones (574* or 575* with any extension) and a concomitant diagnosis of acute pancreatitis (577.0)

eTable 2. Individual comorbidities in the training and experimental cohorts among Medicare beneficiaries who underwent laparoscopic or robotic cholecystectomy

eTable 3. Overall patient characteristics in experimental cohort based on operative approach

eTable 4. Low-risk patient characteristics in experimental cohort based on operative approach

eTable 5. Medium-risk patient characteristics in experimental cohort based on operative approach

eTable 6. High-risk patient characteristics in experimental cohort based on operative approach

eTable 7. Bile duct injury rates in training cohort, stratified by operative approach and patient risk factor terciles as described in methods (low, medium, high risk). Relative risks comparing robotic-assisted versus laparoscopic cholecystectomy

eTable 8. Rates of 90-day composite adverse outcomes (comprised of any complications, serious complications, reoperations, and readmissions) within training cohort and stratified by operative approach and patient risk factor terciles as described in methods (low, medium, high risk)

eTable 9. Overall rates of composite outcome measures in training cohort based on patient risk stratification and operative approach for cholecystectomy among Medicare beneficiaries

This supplemental material has been provided by the authors to give readers additional information about their work.

© 2025 Mullens CL et al. *JAMA Network Open*.

eTable 1. Coding strategy for covariates in our primary analysis. Note: Gallstone pancreatitis included any record with any discharge diagnosis code for gallstones (574* or 575* with any extension) and a concomitant diagnosis of acute pancreatitis (577.0)

Variable	CPT/ICD-9/ICD-10 codes
Cholecystectomy	CPT: 49310, 56340, 56342, 47562, 47564, 47600, 47610, 47612, 47620
Cholecystectomy with intraoperative cholangiogram	CPT: 49311, 56341, 47563, 47605
Hepatobiliary, pancreatic, or duodenal malignancy	ICD-9: 152*, 155*, 156*, 157*, 158*, 159*
Biliary colic and/or dyskinesia	ICD-9: 574.2*, 789.00, 789.01, 574.50, 574.70, 575.8, 5752, 5753, 5754, 5755, 5756, 5758, 5759, 576, 5761, 5762, 5763, 5764, 5765, 5768, 5769, ICD-10: K82, K820, K821, K822, K823, K824, K828, K829, K83, K830, K831, K832, K833, K834, K835, K838, K839
Gallstone pancreatitis / common duct stone	ICD-9: 574.3* -574.9*/ 577.0
Cholecystitis	ICD-9: 5740, 57400, 57401, 57410, 57411, 5743, 57430, 57431, 57440, 57441, 5746, 57460, 57461, 57470, 57471, 5748, 57480, 57481, 575, 5750, 5751, 57511, 57512, 57510 ICD-10: K81.0, K81.1, K81.2, K81.9, K800, K8000, K8001, K801, K8010, K8011, K8012, K8013, K8018, K8019, K804, K8040, K8041, K8042, K8043, K8044, K8045, K8046, K8047, K806, K8060, K8061, K8062, K8063, K8064, K8065, K8066, K8067, K810, K811, K812, K819
Cholelithiasis	ICD-9 - 5742, 57420, 57421, 57441 ICD-10 - K80, K82, K83, K802, K8020, K8021, K808, K8080, K8081
Gallstone pancreatitis	ICD-9: 5742, 57420, 57421, 57441, 5770 ICD-10: K85.10, K85.11, K85.12
Gallbladder and hepatopancreaticobiliary cancer	ICD-9: 156.0, 156.1, 156.2, 156.8, 156.9,155.1, 157 ICD-10: C22.1 C22.8 C22.9 C23 C24 C24.0 C24.1 C24.9 C25.0
Laparoscopic cholecystectomy	CPT:49310, 49311, 47562, 47563, 47564, 56340, 56341, 56342
Robotic identifier codes	ICD-9: 17.41-17.44, 17.49 ICD-10: 8E0W0CZ, 8E0W3CZ, 8E0W4CZ, 8E0W7CZ, 8E0W8CZ
Choledochojejunostomy/hepaticojejunostomy	ICD-9: 51.94, 51.36, 51.37, 51.39, 51.79, 51.72 ICD10:0F15 0F16 0F17 0F19 CPT:47701, 47720, 47721, 47740, 47760, 47765, 47780 , 47785

© 2025 Mullens CL et al. *JAMA Network Open*.

eTable 2. Individual comorbidities in the training and experimental cohorts among Medicare beneficiaries who underwent laparoscopic or robotic cholecystectomy

	**Training cohort, No. (%)**	**Experimental cohort, No. (%)**	**p-value**
Congestive heart failure	59,547 (13.5)	39,826 (13.5)	0.95
Valvular disease	32,985 (7.5)	22,117 (7.5)	0.80
Pulmonary circulation disease	7,874 (1.8)	5,212 (1.8)	0.54
Peripheral vascular disease	29,910 (6.8)	19,678 (6.7)	0.06
Hypertension	329,583 (74.5)	220,244 (74.5)	0.36
Paralysis	9,682 (2.2)	6,532 (2.2)	0.60
Other neurological disorders	34,128 (7.7)	22,651 (7.7)	0.33
Chronic pulmonary disease	85,406 (19.3)	56,912 (19.2)	0.40
Diabetes without chronic complications	94,630 (21.4)	62,794 (21.2)	0.07
Diabetes with chronic complications	43,614 (9.9)	29,298 (9.9)	0.58
Hypothyroidism	76,259 (17.2)	51,153 (17.3)	0.63
Renal failure	65,220 (14.8)	43,688 (14.8)	0.84
Liver disease	34,718 (7.9)	23,498 (7.9)	0.16
Peptic ulcer Disease	4,489 (1.0)	2,908 (1.0)	0.17
Acquired immune deficiency syndrome	283 (0.1)	199 (0.1)	0.59
Lymphoma	3,485 (0.8)	2,346 (0.8)	0.82
Metastatic cancer	5,625 (1.3)	3,714 (1.3)	0.53
Solid tumor w/out metastasis	7,601 (1.7)	5,037 (1.7)	0.59
Rheumatoid arthritis/collagen vas	14,781 (3.3)	9,925 (3.4)	0.78
Coagulopathy	25,514 (5.8)	17,182 (5.8)	0.50
Weight loss	26,158 (5.9)	17,398 (5.9)	0.53
Fluid and electrolyte disorders	142,660 (32.3)	95,157 (32.2)	0.37
Chronic blood loss anemia	2,466 (0.6)	1,666 (0.6)	0.76
Deficiency Anemias	69,090 (15.6)	46,233 (15.6)	0.98
Alcohol abuse	6,166 (1.4)	4,012 (1.4)	0.17
Drug abuse	2,911 (0.7)	1,836 (0.6)	0.05
Psychoses	10,297 (2.3)	7,055 (2.4)	0.12
Depression	44,372 (10.0)	29,456 (10.0)	0.27
Obesity	74,459 (16.8)	49,481 (16.7)	0.20

© 2025 Mullens CL et al. *JAMA Network Open*.

eTable 3. Overall patient characteristics in experimental cohort based on operative approach

**Patient characteristics**	**All, No. (**		
	Laparoscopic	Robotic	p-value
Number of patients in cohort	283,522	10,948	
Age in years, mean (SD)	74.7 (9.9)	74.1 (9.7)	<0.001
Female	148,763 (52.5)	5,693 (52.0)	0.33
Race/Ethnicity			<0.001
Asian	5,234 (1.8)	229 (2.1)	
Black	19,839 (7.0)	903 (8.2)	
Hispanic	7,930 (2.8)	306 (2.8)	
North American Native	2,165 (0.8)	62 (0.6)	
White	240,720 (84.9)	9,076 (82.9)	
Other	4,709 (1.7)	210 (1.9)	
Unknown	2,925 (1.0)	162 (1.5)	
Length of stay (days), mean (SD)	5.4 (5.5)	5.6 (5.8)	<0.001
Number of diagnoses during admission, mean (SD)	12.5 (6.2)	14.5 (6.5)	<0.001
Number of surgical procedure count, mean (SD)	2.7 (2.0)	4.0 (2.4)	<0.001
Number of Elixhauser Comorbidities			<0.001
0	17,757 (6.3)	593 (5.4)	
1	46,180 (16.3)	1,516 (13.8)	
2	61,326 (21.6)	2,141 (19.6)	
3	56,891 (20.1)	2,195 (20.0)	
4	42,451 (15.0)	1,775 (16.2)	
5+	58,917 (20.8)	2,728 (24.9)	
Indication for cholecystectomy			
Cholecystitis	249,169 (87.9)	8,746 (79.9)	<0.001
Cholangitis	12,200 (4.3)	507 (4.6)	0.10
Cholelithiasis	18,533 (6.5)	1,073 (9.8)	<0.001
Other	46,026 (16.2)	2,002 (18.3)	<0.001
90-days Composite outcome components			
Any complications	73,349 (25.9)	3,156 (28.8)	<0.001
Serious complications	29,930 (10.6)	1,345 (12.3)	<0.001
Reoperations	9,974 (3.5)	568 (5.2)	<0.001
Readmissions	56,294 (19.9)	2,212 (20.2)	0.37
Elixhauser comorbidities			
Congestive heart failure	38,066 (13.4)	1,586 (14.5)	0.001
Valvular disease	21,175 (7.5)	870 (7.9)	0.06
Pulmonary circulation disease	5,079 (1.8)	115 (1.1)	<0.001
Peripheral vascular disease	18,886 (6.7)	719 (6.6)	0.70
Hypertension	210,953 (74.4)	8,350 (76.3)	<0.001
Paralysis	6,243 (2.2)	267 (2.4)	0.10
Other neurological disorders	21,694 (7.7)	858 (7.8)	0.47
Chronic pulmonary disease	54,559 (19.2)	2,112 (19.3)	0.90
Diabetes w/o chronic complications	60,548 (21.4)	1,977 (18.1)	<0.001
Diabetes w/ chronic complications	27,525 (9.7)	1,647 (15.0)	<0.001
Hypothyroidism	48,901 (17.2)	2,025 (18.5)	<0.001
Renal failure	41,892 (14.8)	1,611 (14.7)	0.86
Liver disease	22,183 (7.8)	1,219 (11.1)	<0.001
Peptic ulcer Disease x bleeding	2,703 (1.0)	191 (1.7)	<0.001
Acquired immune deficiency syndrome	190 (0.1)	< 11 (0.1)	0.63

© 2025 Mullens CL et al. *JAMA Network Open*.

Lymphoma	2,235 (0.8)	99 (0.9)	0.18
Metastatic cancer	3,446 (1.2)	253 (2.3)	<0.001
Solid tumor w/out metastasis	4,763 (1.7)	255 (2.3)	<0.001
Rheumatoid arthritis/collagen vas	9,499 (3.4)	393 (3.6)	0.17
Coagulopathy	16,427 (5.8)	692 (6.3)	0.02
Weight loss	16,544 (5.8)	780 (7.1)	<0.001
Fluid and electrolyte disorders	90,930 (32.1)	3,791 (34.6)	<0.001
Chronic blood loss anemia	1,585 (0.6)	74 (0.7)	0.11
Deficiency Anemias	44,062 (15.5)	1,982 (18.1)	<0.001
Alcohol abuse	3,820 (1.3)	176 (1.6)	0.02
Drug abuse	1,733 (0.6)	96 (0.9)	<0.001
Psychoses	6,760 (2.4)	273 (2.5)	0.46
Depression	28,196 (9.9)	1,149 (10.5)	0.06
Obesity	46,991 (16.6)	2,283 (20.9)	<0.001

© 2025 Mullens CL et al. *JAMA Network Open*.

eTable 4. Low-risk patient characteristics in experimental cohort based on operative approach

**Patient characteristics**	**Low risk, No. (%)**		
	Laparoscopic	Robotic	p-value
Number of patients in cohort	95,793	2,580	
Age in years, mean (SD)	72.7 (9.6)	72.0 (9.6)	<0.001
Female	53,085 (55.4)	1,488 (57.7)	0.02
Race/Ethnicity			<0.001
Asian	1,742 (1.8)	59 (2.3)	
Black	5,336 (5.6)	173 (6.7)	
Hispanic	2,931 (3.1)	75 (2.9)	
North American Native	711 (0.7)	13 (0.5)	
White	82,124 (85.7)	2,144 (83.1)	
Other	1,736 (1.8)	60 (2.3)	
Unknown	1,213 (1.3)	56 (2.2)	
Length of stay (days), mean (SD)	2.5 (1.3)	2.3 (1.2)	<0.001
Number of diagnoses during admission, mean (SD)	7.1 (3.0)	7.3 (2.9)	0.01
Number of surgical procedure count, mean (SD)	1.8 (0.9)	2.8 (1.0)	<0.001
Number of Elixhauser Comorbidities			0.13
0	14,595 (15.2)	443 (17.2)	
1	30,926 (32.3)	859 (33.3)	
2	28,614 (29.9)	747 (29.0)	
3	14,991 (15.6)	371 (14.4)	
4	5,159 (5.4)	123 (4.8)	
5+	1,508 (1.6)	37 (1.4)	
Indication for cholecystectomy			
Cholecystitis	84,898 (88.6)	2,045 (79.3)	<0.001
Cholangitis	2,689 (2.8)	68 (2.6)	0.60
Cholelithiasis	6,536 (6.8)	303 (11.7)	<0.001
Other	11,414 (11.9)	351 (13.6)	0.01
90-days Composite outcome components			
Any complications	6,076 (6.3)	156 (6.0)	0.54
Serious complications	765 (0.8)	8 (0.3)	0.01
Reoperations	1,767 (1.8)	70 (2.7)	0.001
Readmissions	10,953 (11.4)	258 (10.0)	0.02
Elixhauser comorbidities			
Congestive heart failure	492 (0.5)	< 11 (0.2)	0.05
Valvular disease	3,033 (3.2)	83 (3.2)	0.88
Pulmonary circulation disease	441 (0.5)	< 11 (0.2)	0.02
Peripheral vascular disease	2,820 (2.9)	56 (2.2)	0.02
Hypertension	61,759 (64.5)	1,549 (60.0)	<0.001
Paralysis	818 (0.9)	18 (0.7)	0.39
Other neurological disorders	4,175 (4.4)	106 (4.1)	0.54
Chronic pulmonary disease	12,512 (13.1)	263 (10.2)	<0.001
Diabetes w/o chronic complications	18,885 (19.7)	456 (17.7)	0.01
Diabetes w/ chronic complications	1,294 (1.4)	43 (1.7)	0.17
Hypothyroidism	13,654 (14.3)	358 (13.9)	0.59
Renal failure	62 (0.1)	< 11 (0.0)	0.61
Liver disease	5,181 (5.4)	215 (8.3)	<0.001
Peptic ulcer Disease x bleeding	326 (0.3)	16 (0.6)	0.02
Acquired immune deficiency syndrome	52 (0.1)	< 11 (0.1)	0.62

© 2025 Mullens CL et al. *JAMA Network Open*.

Lymphoma	526 (0.5)	13 (0.5)	0.76
Metastatic cancer	590 (0.6)	24 (0.9)	0.05
Solid tumor w/out metastasis	1,077 (1.1)	44 (1.7)	0.01
Rheumatoid arthritis/collagen vas	2,411 (2.5)	63 (2.4)	0.81
Coagulopathy	1,555 (1.6)	35 (1.4)	0.29
Weight loss	274 (0.3)	5 (0.2)	0.38
Fluid and electrolyte disorders	5,865 (6.1)	133 (5.2)	0.04
Chronic blood loss anemia	130 (0.1)	2 (0.1)	0.43
Deficiency Anemias	2,863 (3.0)	65 (2.5)	0.17
Alcohol abuse	690 (0.7)	26 (1.0)	0.09
Drug abuse	331 (0.3)	< 11 (0.3)	0.98
Psychoses	1,680 (1.8)	49 (1.9)	0.58
Depression	6,756 (7.1)	170 (6.6)	0.36
Obesity	11,364 (11.9)	337 (13.1)	0.06

© 2025 Mullens CL et al. *JAMA Network Open*.

eTable 5. Medium-risk patient characteristics in experimental cohort based on operative approach

Patient characteristics	Medium risk, No. (%)		
	Laparoscopic	Robotic	p-value
Number of patients in cohort	94,351	3,778	
Age in years, mean (SD)	75.1 (9.7)	73.6 (9.5)	<0.001
Female	49,964 (53.0)	2,032 (53.8)	0.32
Race/Ethnicity			0.02
Asian	1,671 (1.8)	70 (1.9)	
Black	6,025 (6.4)	262 (6.9)	
Hispanic	2,434 (2.6)	102 (2.7)	
North American Native	688 (0.7)	19 (0.5)	
White	81,068 (85.9)	3,201 (84.7)	
Other	1,497 (1.6)	65 (1.7)	
Unknown	968 (1.0)	59 (1.6)	
Length of stay (days), mean (SD)	4.4 (2.1)	3.9 (1.9)	<0.001
Number of diagnoses during admission, mean (SD)	12.2 (3.9)	12.8 (4.0)	<0.001
Number of surgical procedure count, mean (SD)	2.4 (1.4)	3.5 (1.5)	<0.001
Number of Elixhauser Comorbidities			<0.001
0	2,612 (2.8)	132 (3.5)	
1	12,572 (13.3)	570 (15.1)	
2	24,739 (26.2)	1,029 (27.2)	
3	26,604 (28.2)	1,047 (27.7)	
4	17,081 (18.1)	621 (16.4)	
5+	10,743 (11.4)	379 (10.0)	
Indication for cholecystectomy			
Cholecystitis	82,150 (87.1)	2,961 (78.4)	<0.001
Cholangitis	4,735 (5.0)	181 (4.8)	0.53
Cholelithiasis	6,517 (6.9)	400 (10.6)	<0.001
Other	16,117 (17.1)	651 (17.2)	0.81
90-days Composite outcome components			
Any complications	17,755 (18.8)	610 (16.1)	<0.001
Serious complications	2,192 (2.3)	68 (1.8)	0.04
Reoperations	3,040 (3.2)	183 (4.8)	<0.001
Readmissions	16,594 (17.6)	566 (15.0)	<0.001
Elixhauser comorbidities			
Congestive heart failure	7,299 (7.7)	198 (5.2)	<0.001
Valvular disease	6,551 (6.9)	234 (6.2)	0.08
Pulmonary circulation disease	1,272 (1.3)	27 (0.7)	<0.001
Peripheral vascular disease	5,920 (6.3)	197 (5.2)	0.008
Hypertension	72,460 (76.8)	2,882 (76.3)	0.46
Paralysis	1,725 (1.8)	60 (1.6)	0.28
Other neurological disorders	7,247 (7.7)	241 (6.4)	0.003
Chronic pulmonary disease	18,417 (19.5)	690 (18.3)	0.06
Diabetes w/o chronic complications	22,612 (24.0)	793 (21.0)	<0.001
Diabetes w/ chronic complications	6,049 (6.4)	285 (7.5)	0.006
Hypothyroidism	16,896 (17.9)	686 (18.2)	0.69
Renal failure	5,742 (6.1)	125 (3.3)	<0.001
Liver disease	8,071 (8.6)	449 (11.9)	<0.001
Peptic ulcer Disease x bleeding	819 (0.9)	47 (1.2)	0.02

© 2025 Mullens CL et al. *JAMA Network Open*.

Acquired immune deficiency syndrome	57 (0.1)	< 11 (0.1)	0.85
Lymphoma	718 (0.8)	22 (0.6)	0.21
Metastatic cancer	1,108 (1.2)	94 (2.5)	<0.001
Solid tumor w/out metastasis	1,587 (1.7)	73 (1.9)	0.24
Rheumatoid arthritis/collagen vas	3,303 (3.5)	132 (3.5)	0.98
Coagulopathy	4,463 (4.7)	162 (4.3)	0.21
Weight loss	2,733 (2.9)	92 (2.4)	0.10
Fluid and electrolyte disorders	30,401 (32.2)	996 (26.4)	<0.001
Chronic blood loss anemia	440 (0.5)	18 (0.5)	0.93
Deficiency Anemias	11,601 (12.3)	407 (10.8)	0.005
Alcohol abuse	1,420 (1.5)	56 (1.5)	0.91
Drug abuse	582 (0.6)	46 (1.2)	<0.001
Psychoses	2,316 (2.5)	92 (2.4)	0.94
Depression	10,033 (10.6)	413 (10.9)	0.56
Obesity	16,248 (17.2)	784 (20.8)	<0.001

© 2025 Mullens CL et al. *JAMA Network Open*.

eTable 6. High-risk patient characteristics in experimental cohort based on operative approach

**Patient characteristics**	**High risk, No. (%)**		
	Laparoscopic	Robotic	p-value
Number of patients in cohort	93,378	4,590	
Age in years, mean (SD)	76.4 (10.0)	75.6 (9.7)	<0.001
Female	45,714 (49.0)	2,173 (47.3)	0.03
Race/Ethnicity			0.02
Asian	1,821 (2.0)	100 (2.2)	
Black	8,478 (9.1)	468 (10.2)	
Hispanic	2,565 (2.7)	129 (2.8)	
North American Native	766 (0.8)	30 (0.7)	
White	77,528 (83.0)	3,731 (81.3)	
Other	1,476 (1.6)	85 (1.9)	
Unknown	744 (0.8)	47 (1.0)	
Length of stay (days), mean (SD)	9.4 (7.8)	8.9 (7.6)	<0.001
Number of diagnoses during admission, mean (SD)	18.4 (5.2)	19.9 (4.6)	<0.001
Number of surgical procedure count, mean (SD)	3.8 (2.7)	5.0 (3.1)	<0.001
Number of Elixhauser Comorbidities			0.002
0	550 (0.6)	18 (0.4)	
1	2,682 (2.9)	87 (1.9)	
2	7,973 (8.5)	365 (8.0)	
3	15,296 (16.4)	777 (16.9)	
4	20,211 (21.6)	1,031 (22.5)	
5+	46,666 (50.0)	2,312 (50.4)	
Indication for cholecystectomy			
Cholecystitis	82,121 (87.9)	3,740 (81.5)	<0.001
Cholangitis	4,776 (5.1)	258 (5.6)	0.13
Cholelithiasis	5,480 (5.9)	370 (8.1)	<0.001
Other	18,495 (19.8)	1,000 (21.8)	0.001
90-days Composite outcome components			
Any complications	49,518 (53.0)	2,390 (52.1)	0.20
Serious complications	26,973 (28.9)	1,269 (27.6)	0.07
Reoperations	5,167 (5.5)	315 (6.9)	<0.001
Readmissions	28,747 (30.8)	1,388 (30.2)	0.43
Elixhauser comorbidities			
Congestive heart failure	30,275 (32.4)	1,382 (30.1)	0.001
Valvular disease	11,591 (12.4)	553 (12.0)	0.46
Pulmonary circulation disease	3,366 (3.6)	84 (1.8)	<0.001
Peripheral vascular disease	10,146 (10.9)	466 (10.2)	0.13
Hypertension	76,734 (82.2)	3,919 (85.4)	<0.001
Paralysis	3,700 (4.0)	189 (4.1)	0.60
Other neurological disorders	10,272 (11.0)	511 (11.1)	0.78
Chronic pulmonary disease	23,630 (25.3)	1,159 (25.3)	0.93
Diabetes w/o chronic complications	19,051 (20.4)	728 (15.9)	<0.001
Diabetes w/ chronic complications	20,182 (21.6)	1,319 (28.7)	<0.001
Hypothyroidism	18,351 (19.7)	981 (21.4)	0.004
Renal failure	36,088 (38.6)	1,485 (32.4)	<0.001
Liver disease	8,931 (9.6)	555 (12.1)	<0.001
Peptic ulcer Disease x bleeding	1,558 (1.7)	128 (2.8)	<0.001
Acquired immune deficiency syndrome	81 (0.1)	< 11 (0.0)	0.33

© 2025 Mullens CL et al. *JAMA Network Open*.

Lymphoma	991 (1.1)	64 (1.4)	0.03
Metastatic cancer	1,748 (1.9)	135 (2.9)	<0.001
Solid tumor w/out metastasis	2,099 (2.2)	138 (3.0)	0.001
Rheumatoid arthritis/collagen vas	3,785 (4.1)	198 (4.3)	0.38
Coagulopathy	10,409 (11.1)	495 (10.8)	0.45
Weight loss	13,537 (14.5)	683 (14.9)	0.47
Fluid and electrolyte disorders	54,664 (58.5)	2,662 (58.0)	0.46
Chronic blood loss anemia	1,015 (1.1)	54 (1.2)	0.57
Deficiency Anemias	29,598 (31.7)	1,510 (32.9)	0.09
Alcohol abuse	1,710 (1.8)	94 (2.0)	0.29
Drug abuse	820 (0.9)	41 (0.9)	0.91
Psychoses	2,764 (3.0)	132 (2.9)	0.74
Depression	11,407 (12.2)	566 (12.3)	0.82
Obesity	19,379 (20.8)	1,162 (25.3)	<0.001

© 2025 Mullens CL et al. *JAMA Network Open*.

eTable 7. Bile duct injury rates in training cohort, stratified by operative approach and patient risk factor terciles as described in methods (low, medium, high risk). Relative risks comparing robotic-assisted versus laparoscopic cholecystectomy

	**Robotic-Assisted**	**Laparoscopic**	**Relative Risk (95% CI)**
	Mean (95% CIs)	*Mean (95% CIs)*	
Low risk	0.42 (0.32-0.53)	0.17 (0.15-0.19)	2.54 (1.95-3.12)
Medium risk	0.57 (0.43-0.70)	0.22 (0.20-0.25)	2.53 (1.95-3.11)
High risk	0.81 (0.62-0.99)	0.32 (0.29-0.35)	2.52 (1.94-3.09)
Overall	0.60 (0.47-0.73)	0.24 (0.22-0.25)	2.53 (1.95-3.11)

© 2025 Mullens CL et al. *JAMA Network Open*.

eTable 8. Rates of 90-day composite adverse outcomes (comprised of any complications, serious complications, reoperations, and readmissions) within training cohort and stratified by operative approach and patient risk factor terciles as described in methods (low, medium, high risk)

	**Robotic-Assisted**	**Laparoscopic**
	Mean (95% CIs)	*Mean (95% CIs)*
Low risk	0.20 (0.19-0.21)	0.17 (0.16-0.18)
Medium risk	0.50 (0.49-0.51)	0.50 (0.49-0.51)
High risk	1.16 (1.15-1.17)	1.13 (1.12-1.14)
Overall	0.69 (0.68-0.71)	0.60 (0.59-0.61)

© 2025 Mullens CL et al. *JAMA Network Open*.

eTable 9. Overall rates of composite outcome measures in training cohort based on patient risk stratification and operative approach for cholecystectomy among Medicare beneficiaries. Relative risks comparing robotic-assisted versus laparoscopic cholecystectomy

90-day outcomes (yes/no)	**Robotic-Assisted**	**Laparoscopic**	**Relative Risk (95% CI)**
	Mean (95% CIs)	*Mean (95% CIs)*	
Low Risk			
Composite outcome	15.39 (14.19-16.59)	16.06 (15.87-16.25)	0.96 (0.88-1.03)
Any complications	6.11 (5.31-6.91)	6.35 (6.22-6.48)	0.96 (0.84-1.09)
Serious complications	0.63 (0.37-0.90)	0.80 (0.75-0.84)	0.79 (0.46-1.13)
Reoperations	3.29 (2.69-3.88)	1.92 (1.85-2.00)	1.71 (1.39-2.02)
Readmissions	9.91 (8.92-10.91)	11.47 (11.31-11.64)	0.86 (0.78-0.95)
Medium Risk			
Composite outcome	29.59 (28.32-30.86)	31.64 (31.39-31.88)	0.94 (0.89-0.98)
Any complications	16.03 (15.01-17.06)	18.90 (18.70-19.11)	0.85 (0.79-0.90)
Serious complications	2.02 (1.63-2.41)	2.32 (2.24-2.39)	0.87 (0.70-1.04)
Reoperations	5.89 (5.23-6.55)	3.24 (3.15-3.34)	1.82 (1.61-2.02)
Readmissions	14.99 (13.99-15.98)	17.40 (17.20-17.60)	0.86 (0.80-0.92)
High Risk			
Composite outcome	62.86 (61.61-64.10)	64.39 (64.14-64.64)	0.98 (0.96-1.00)
Any complications	51.23 (49.94-52.53)	52.80 (52.54-53.06)	0.97 (0.95-1.00)
Serious complications	26.13 (24.99-27.26)	28.98 (28.74-29.22)	0.90 (0.86-0.94)
Reoperations	7.68 (6.99-8.37)	5.29 (5.17-5.41)	1.45 (1.32-1.59)
Readmissions	28.16 (27.00-29.32)	30.67 (30.42-30.91)	0.92 (0.88-0.96)
Overall			
Composite outcome	39.62 (38.81-40.42)	37.27 (37.12-37.41)	1.06 (1.04-1.08)
Any complications	27.88 (27.14-28.61)	25.93 (25.80-26.06)	1.08 (1.05-1.10)
Serious complications	11.44 (10.92-11.97)	10.65 (10.56-10.74)	1.07 (1.02-1.12)
Reoperations	5.99 (5.60-6.38)	3.48 (3.43-3.54)	1.72 (1.61-1.84)
Readmissions	19.11 (18.46-19.75)	19.82 (19.70-19.93)	0.96 (0.93-1.00)

© 2025 Mullens CL et al. *JAMA Network Open*.
