# Supplementary material for: Patient Complexity and Bile Duct Injury After Robotic-Assisted vs Laparoscopic Cholecystectomy
Source: JAMA Netw Open. 2025 Mar 25;8(3):e251705. doi: 10.1001/jamanetworkopen.2025.1705 (PMC11937934; doi:10.1001/jamanetworkopen.2025.1705)
Supplement: Supplement 2. — Data Sharing Statement [file jamanetwopen-e251705-s002.pdf]

Data Sharing Statement

Mullens. Patient Complexity and Bile Duct Injury After Robotic-Assisted vs Laparoscopic Cholecystectomy. *JAMA Netw Open*. Published March 25, 2025.
doi:10.1001/jamanetworkopen.2025.1705

Data
Data available: No

Additional Information
Explanation for why data not available: Due to data use agreement with Medicare.
